# Investigation and Comparison of Active and Passive Encapsulation Methods for Loading Proteins into Liposomes

**DOI:** 10.3390/ijms241713542

**Published:** 2023-08-31

**Authors:** Silvia Pisani, Deborah Di Martino, Silvia Cerri, Ida Genta, Rossella Dorati, Giulia Bertino, Marco Benazzo, Bice Conti

**Affiliations:** 1 Department of Drug Sciences, University of Pavia, Viale T. Taramelli 12, 27100 Pavia, Italy; silvia.pisani@unipv.it (S.P.); ida.genta@unipv.it (I.G.); rossella.dorati@unipv.it (R.D.); 2 Unit of Cellular and Molecular Neurobiology, IRCCS Mondino Foundation, Via Mondino 2, 27100 Pavia, Italy; deborah.dimartino@mondino.it (D.D.M.); silvia.cerri@mondino.it (S.C.); 3 Otorhinolaryngology Unit, Department of Surgical Sciences, Fondazione IRCCS Policlinico San Matteo, 27100 Pavia, Italy; g.bertino@smatteo.pv.it (G.B.); m.benazzo@smatteo.pv.it (M.B.)

**Keywords:** liposomes, microfluidic, freeze–thawing, electroporation, bovine serum albumin

## Abstract

In this work, four different active encapsulation methods, microfluidic (MF), sonication (SC), freeze–thawing (FT), and electroporation (EP), were investigated to load a model protein (bovine serum albumin—BSA) into neutral liposomes made from 1,2-distearoyl-sn-glycero-3-phosphocholine (DSPC):cholesterol (Chol) and charged liposomes made from DSPC:Chol:Dioleoyl-3-trimethylammonium propane (DOTAP), DSPC:Chol:1,2-dioleoyl-sn-glycero-3-phospho-L-serine (DOPS), and DSPC:Chol:phosphatidylethanolamine (PE). The aim was to increase the protein encapsulation efficiency (EE%) by keeping the liposome size below 200 nm and the PDI value below 0.7, which warrants a nearly monodisperse preparation. Electroporation (100 V) yielded the best results in terms of EE%, with a dramatic increase in liposome size (>600 nm). The FT active-loading method, either applied to neutral or charged liposomes, allowed for obtaining suitable EE%, keeping the liposome size range below 200 nm with a suitable PDI index. Cationic liposomes (DSPC:Chol:DOTAP) loaded with the FT active method showed the best results in terms of EE% (7.2 ± 0.8%) and size (131.2 ± 11.4 nm, 0.140 PDI). In vitro release of BSA from AM neutral and charged liposomes resulted slower compared to PM liposomes and was affected by incubation temperature (37 °C, 4 °C). The empty charged liposomes tested for cell viability on Human Normal Dermal Fibroblast (HNDF) confirmed their cytocompatibility also at high concentrations (10^10^ particles/mL) and cellular uptake at 4 °C and 37 °C. It can be concluded that even if both microfluidic passive and active methods are more easily transferable to an industrial scale, the FT active-loading method turned out to be the best in terms of BSA encapsulation efficiencies, keeping liposome size below 200 nm.

## 1. Introduction

Liposomes are colloidal vesicles made from cholesterol and other lipids and having properties that make them biocompatible, biodegradable, and non-toxic. The use of liposomes permits the increase in the therapeutic index of many drugs and also achieves drug targeting and controlled release [1]. Liposome behavior is influenced by composition and size, as reported in the extensive literature on this topic [2,3,4]. As far as liposome composition is concerned, cholesterol increases liposome stability and performance because its blending with other lipids leads the molecule to distribute with its hydroxyl group close to the head lipids’ group region while the aromatic rings align with the hydrophobic alkyl chains. This configuration causes increased fluidity in the liposome bilayer core but an increase in the viscosity and rigidity in the headgroups’ region with increased liposome stability and decreased interaction with plasma proteins. Eventually, the presence of cholesterol favors the loading of hydrophilic drugs into the liposome core. Lipid composition is crucial in determining liposome behavior. Liposome fluidity changes depending on the lipid’s main transition temperature (Tm) value, and liposomes containing lipids with high Tm, such as the saturated phospholipid Distearoylphosphatidylcholine (DSPC), result in a more rigid and stable bilayer structure with a greater ability to retain the encapsulated drugs [2,3]. Moreover, lipid composition can be selected to achieve liposome surface net charge and modulate their interaction with biologic environment (cells). In these cases, cationic, anionic, or ionizable lipids are blended in the lipidic composition of liposomes, depending on the liposome target. In general, surface charge ameliorates liposome stability by preventing aggregation effects through electrostatic repulsion among liposomes [5]. Negatively charged liposomes are easily uptaken by macrophages and enter the cells through endocytosis. Cationic liposomes interact with glycoproteins of endothelial cell membranes and show longer circulation half-lives with respect to anionic liposomes. They also interact with the anionic components of the blood (such as plasma proteins), and their uptake by the mononuclear phagocytic system (MPS) leads them to be cleared by the liver, lung, or spleen [3]. Cationic liposomes are preferred for gene delivery because of their ability to make complexes with anionic nucleic acids [4]. In the pharmaceutical area, liposome size ranges between 50 and 500 nm, and it affects the drug delivery process. Liposomes with diameters in the range of 100–200 nm are better uptaken by cells and, when injected in vivo, can undergo enhance permeability and retention (EPR) effects and enter through the fenestrated vessels into tumor environments [2,6,7]. Liposomes with diameters in the range of 50–100 nm or less can avoid immune system phagocytosis clearance and exhibit longer blood circulation times [8,9]. Therefore, liposome size is an important parameter to be considered when designing a liposome formulation, and it is affected by the preparation and drug loading method [10,11]. The poly dispersity index (PDI) value provides information about the dispersion of liposome size in a size range. A PDI value of 0.1 to 0.7 represents nearly monodisperse preparation; thus, the closer this value is to zero, the more monodisperse the preparation. It is well known that PDI is also affected by the liposome preparation methods [12].

Protein-based therapeutics have found widespread applications in the treatment of cancer, infectious diseases, and other metabolic disorders, and protein loading into liposomes, among the diverse advantages, can contribute to overcoming protein diffusion, improve its bioavailability and stability [13]. In fact, the bonds involved in the tertiary structure of proteins related to their biological action are weak interactions, and proteins are subjected to in vivo degradation by proteases [14].

Protein loading inside liposomes is challenging because of the proteins’ high molecular weight and their three-dimensional structure, as investigated by other authors and reported in the literature [15,16]. The process can be performed through passive or active-loading methods; also, a combination of the two methods can be used. Briefly, the passive method involves protein internalization during the liposome assembly and formation steps. The active-loading method, also called remote drug loading, involves filling the preformed liposomes with drug molecules that are able to diffuse one way only [17]. Active loading (also called remote loading) is an alternative strategy for drug encapsulation that takes advantage of transmembrane chemical gradients to entrap amphipathic compounds from the surrounding environment into preformed liposomes [18]. Usually, active loading is based on concentration gradient loading techniques involving buffer or ammonium sulfate gradients [19]. The liposome preparation methods can affect liposome size and the amount of drug loaded into liposomes. It should be studied and tailored according to the type of protein (or class of proteins) to be encapsulated and is a factor of utmost importance to be set up when designing the liposome manufacturing process [1]. To the best of the authors’ knowledge, no scientific literature was found specifically comparing the passive and active methods of loading proteins into liposomes.

Therefore, starting from this background, this work aimed at exploring and comparing the passive protein-loading method by microfluidics with four methods for the active loading of proteins into DSPC:cholesterol-based preformed liposomes. DSPC was chosen as the lipid component because it is a well-known and experimented lipid used in liposome formulation. For example, DSPC is used in the Moderna COVID-19 vaccine formulation, demonstrating its safety [20]. It has been proven to increase liposome rigidity and stability, meanwhile enhancing encapsulation efficiency and lowering liposomes cytotoxicity compared to other lipids [21,22]. The parameters evaluated and compared were protein encapsulation efficiency (EE%) and liposome size (nm ± SD and PDI). The four techniques were selected as popular nanosystem active-loading methods [17]. The microfluidics method was selected as the passive-loading method, and it was chosen because it is a very convenient, automated, scalable, and reproducible method to prepare nanosized delivery systems such as liposomes [23]. The active-loading process by microfluidic (AM_MF) is driven by a concentration differential between the aqueous solution of protein and the core of liposome suspension. It is a diffusion process that happens at room temperature, thus in mild conditions [18]. Active loading, or forced entrapment of hydrophilic compounds into the liposome core via ion/pH gradients, enables high concentrations of reagents to be stored within the vesicles with excellent long-term stability [24].

The other active methods investigated in this work were: sonication (SC), freeze–thawing (FT), and electroporation (EP). These methods act by perturbing the liposome bilayer in order to permeabilize the liposome membrane and induce protein diffusion in the liposome core.

Sonication (SC, 2–35 kHz) induces temporary membrane permeabilization by applying ultrasound: mechanical disruption is achieved via the process of cavitation. The transient liposome membrane permeabilization promotes hydrophilic compound diffusion into the liposome core. This technique is also used to induce active cargo loading in extracellular vesicles (EVs) [25,26].

Freeze–thawing (FT) cycling is a technique often used to increase liposome encapsulation efficiency [27]. The technique induces permeabilization of the liposomes lipid bilayer and allows the entry of molecules, promoting encapsulation. The procedure commonly involves freezing the liposomes with liquid nitrogen (−196 °C) and thawing them at 37 °C. The drug encapsulation mechanism is due to pore formation in the lipid bilayer caused by ice crystals generated during the freezing cycle and their subsequent fusion during the thawing cycle [28].

Electroporation (EP) exploits the use of short high-voltage pulses (100–500 V) to overcome the barrier of the biological membranes. By applying an external electric field, which just overcomes the capacitance of the membrane, transient and reversible breakdown can be induced. This transient, permeabilized state can be used to load cells or liposomes with a variety of different molecules through the electrophoretically driven processes, allowing passage through the destabilized membrane [26,29,30].

Bovine serum albumin (BSA) was chosen as the model protein. It is a globular protein of 69,324 MW and 4.5 isoelectric point (IP), which could be illustrative of those proteins intended to be delivered as drugs encapsulated into liposomes and having properties similar to BSA.

The focus and novelty of this investigation was to evaluate which of the loading methods was the most convenient from the point of view of efficiency in loading a macromolecule by keeping the liposome size lower than 200 nm. This liposome size was selected because it is well known from the literature that liposomes whose size was below 200 nm suitably interact with cell membranes while avoiding the onset of complement system activation and consequent severe toxicity [12,31]. Moreover, the polydispersity index (PDI) value was addressed to be kept under 0.3, which corresponds to uniform liposome size distribution. This first investigation is a step toward subsequent studies of the active loading of protein and peptide drugs in liposomes or even extracellular vesicles (EVs) due to the similarity of their structure [32].

## 2. Results

### 2.1. Physical-Chemical Characterization of DSPC: Chol Liposomes

**Figure 1:**
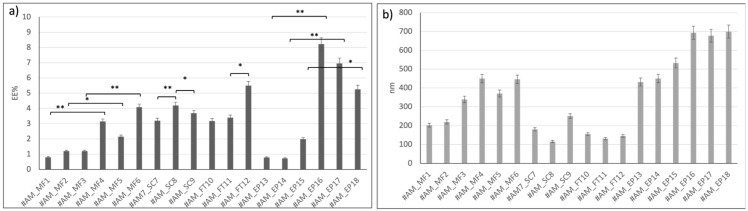
Results of: (**a**) BSA encapsulation efficiency (EE%) and (**b**) particle size of liposomes obtained through active methods. A *p*-value less than 0.05 (* *p* < 0.05) was considered statistically significant, while a *p*-value higher than 0.05 (** *p* >0.05) was considered not statistically significant.

Liposomes prepared using active methods were compared in terms of EE% and size. Figure 1 reports the results obtained.

Liposomes loaded with active method based on microfluidics (from #AM_MF1 to #AM_MF3) showed EE% lower than 2%. These batches were prepared with BSA solubilized in PBS, which is hampered to enter the liposomes due to its dimerization in PBS. Instead, liposomes loaded with the active microfluidic method (from #AM_MF4 to #AM_MF6) and BSA solubilized in MilliQ water (pH 7 by NaOH addition) showed an average EE% between 2.2 ± 0.4 % and 4.1 ± 0.7 %, respectively. So, encapsulation of BSA from PBS solution (#AM_MF1, #AM_MF2, and #AM_MF3) was lower compared to BSA encapsulation from aqueous solution (#AM_MF4, #AM_MF5, and #AM_MF6). Moreover, liposome dimensions increased (>300 nm), increasing the BSA EE%. Consistently, liposomes encapsulating BSA from aqueous solutions showed dimensions > 300 nm.

Liposomes loaded through sonication (#AM_SC7, #AM_SC8, and #AM_SC9) confirmed that BSA encapsulation efficiency is greater when BSA is solubilized in aqueous solutions (#AM_SC8 4.2 ± 0.7 EE%) with respect to BSA in PBS solutions (#AM_SC7 3.2 ± 0.4 EE%). Sonication time resulted in being a parameter affecting liposome size that remained smaller than 200 nm until 36 s sonication time (#AM_SC7 180 ± 18 nm and #AM_SC8 115.6 ± 12 nm). An increase in liposome size (#AM_SC9 250.8 ± 21 nm) was registered by increasing the sonication time to 60 s.

The sonication active method yielded suitable results in terms of BSA EE%, keeping liposome size well below 200 nm (see batch AM_SC8). Thus, the sonication active-loading method does not seem to affect liposome size.

The FT method permitted achieving liposomes (batches #AM_FT10, #AM_FT11, and #AM_FT12) with high EE% and suitable dimensions lower than 200 nm compared to the other active methods tested. The results confirm that BSA encapsulation was favored when the protein was solubilized in aqueous solutions. Moreover, liposome size does not seem to be affected by BSA loading (#AM_FT11 = 3.4 ± 0.3 EE% and 131 ± 15 nm #AM_FT12 = 5.5 ± 0.3 EE% and 145.6 ± 13 nm). Indeed, increasing the number of FT cycles (#AM_FT12), led to increase the EE% without altering final liposomes dimensions. For these reasons, batch #AM_FT12 showed greater results in terms of size (<200 nm), EE% (>5%), and DC% (19.64 ± 0.5%) and was selected for further characterization, and FT was selected as the active method to load BSA into liposomes made from charged and ionizable lipids.

Electroporation was the active-loading method resulting in the highest results in terms of EE% of BSA in aqueous solutions (#AM_EP16 = 8.2 ± 0.9 EE%, #AM_EP17 = 7 ± 1 EE%, and #AM_EP18 = 5.3 ± 0.6 EE%), but it led to a dramatic increase in liposome size, bigger than 600 nm (#AM_EP16 = 693.2 ± 36 nm, #AM_EP17 = 677.4 ± 40 nm, and #AM_EP18 = 699.2 ± 39 nm). Since this liposome size was out of the scope of this work, electroporation was not further investigated as a BSA active-loading method.

In summary, BSA encapsulation by the active-loading method based on microfluidics and electroporation resulted in increasing liposome size above 200 nm, while the SC and FT active-loading methods did not seem to affect liposome size, and FT resulted in the highest EE%.

### 2.2. Physical-Chemical Characterization of Surface-Charged Liposomes

The three types of empty liposomes (cationic #PM5, zwitterionic #PM9, and anionic#PM13) were analyzed by TEM before being used for BSA active encapsulation. 

**Figure 2:**
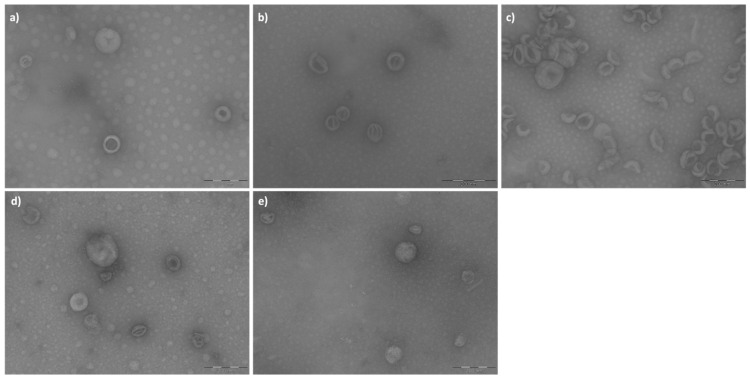
TEM images of batch (**a**) #PM5, (**b**) #PM9, (**c**) #PM13, (**d**) #AM_FT12, and (**e**) #AM_FT19.

The images obtained were processed with ImageJ software 1.53k (Java 13.0.6.), and the results are reported in Table 1. The liposomes of batch #PM13 (Figure 2c) appear less homogeneous in terms of size and shape, and aggregates are observed. The liposomes of batch #PM5 (Figure 2a), on the other hand, appear more spherical and without aggregates. AM_FT19 (Figure 2e, BSA-loaded cationic liposomes) was similar to the corresponding batch PM5 (Figure 2a) empty cationic liposomes as a circularity parameter, but the liposome size was significantly greater than the same results comparing AM_FT19 (Figure 2d,e) with AM_FT12 (BSA-loaded neutral liposomes).

The results of the circularity parameter indicated that in all cases, the liposomes produced have a spherical shape (values close to 1). The liposome dimensions calculated with the ImageJ program are consistent with the liposome size obtained via DLS (Table 1).

The results of characterizations in terms of zeta potential and EE% performed on empty and BSA-loaded charged liposomes are reported in Table 2.

All the charged liposomes obtained using either the passive method (PM) by microfluidic or FT active method (AM_FT) showed dimensions lower than 200 nm and PDIs well below 0.7, demonstrating them to be nearly monodispersed. A 5% molar ratio of charged lipids (cationic, batch #PM5 and anionic, batch #PM13) allowed for obtaining net surface charges (#PM5 +20 ± 3.0 mV and #PM13 −39.3 ± 2.1) and greater EE%, as reported in Table 2. Batch #PM6 is the one that showed the highest EE% (4.57 ± 0.2%) among those batches prepared by the passive method, even higher than that of batch #PM2 (EE% 4.0 ± 1.3%), made from neutral lipids. It can be hypothesized that the EE% increase was due to the positive charge of the liposomes with DOTAP, which favors protein internalization by electrostatic interaction with the negative charges of the BSA (I.P. = 4.9) at pH 7.4. Moreover, PM6 positive zeta potential (+18 ± 1.3 mV), comparable to that of the corresponding empty liposome (batch #PM5), confirms that BSA is preferentially encapsulated into the liposome core. 

As far as zeta potential is concerned, the same behavior is highlighted for anionic liposomes, whose surface charge did not change, comparing empty liposomes batch #PM13 (−39.3 ± 2.1 mV) to BSA-loaded liposomes of batch #PM14 (−41.5 ± 2.7 mV). These results demonstrate that in both cases of cationic and anionic liposomes, the passive-loading method led to preferential protein loading into the liposome core by keeping the liposome size well below 200 nm and PDI values in narrow ranges corresponding to nearly monodispersed preparations (see Table 2).

Positively charged liposomes showed greater results in terms of EE% both with the passive (batch #PM6, 5.57 ± 0.2) and active method (batch AM_FT 19, 7.2 ± 0.8%), and the latter resulted in the greatest EE%. Comparing the results of EE% obtained with neutral lipids (batch #AM_FT12, 5.5 ± 0.3%), positively charged liposomes improved the final EE% by about 25%. The zeta potential of batch AM_FT19 was positive (+16 ± 3.1 mV) even if slightly but significantly lower than the zeta potential of the corresponding empty liposomes batch #PM5 (+20 ± 3.0 mV), demonstrating that BSA is preferentially encapsulated into the liposome core. The liposomes keep suitable size and PDI values (165.8 ± 5.9 nm, PDI = 0.268 ± 0.02) after being loaded with BSA through the FT active-loading method.

Drug content (DC%) values are in trend with the EE% data; therefore, batch #AM_FT19 is the one that, with the same weight (mg) and yield (particle/mL), has the highest protein content (25.71 ± 1.1%). 

As far as liposomes containing zwitterionic lipid PE and BSA are concerned, the surface charge of batch PM9 linearly switches according to aqueous pH media, as expected by the presence of the zwitterionic lipid. It was highly positive at pHs lower than 6 and negative at pHs higher than 8, almost neutral at pH 7 (see Figure S1). This suggests that the liposome surface is not completely coated by BSA. The EE% of batch PM10, AM_FT20, and batch M12 was evaluated using BSA solubilized in pH 7 aqueous solution, and the slight negative surface charge of batch #PM10 (−11.2 ± 1.1) led to EE% values (2.25 ± 0.2%), similar to that recorded for anionic liposomes (PM12, −14.5 ± 3.2 mV, and 2.28 ± 0.4 EE%).

Negatively charged liposomes containing DOPS obtained either by passive-loading or active-loading methods (batches PM 12, PM14, AM_FT21) resulted in the lowest EE%. This behavior is consistent with the liposome negative charge that hampers BSA encapsulation. 

The yield of production expressed as particles/mL was in the range of 10^11^ liposomes/mL for all batches analyzed. In summary, the results obtained show that positively charged lipids improve BSA encapsulation both by passive and active-loading methods by keeping suitable liposome size and PDI values.

### 2.3. BSA In Vitro Release Test

**Figure 3:**
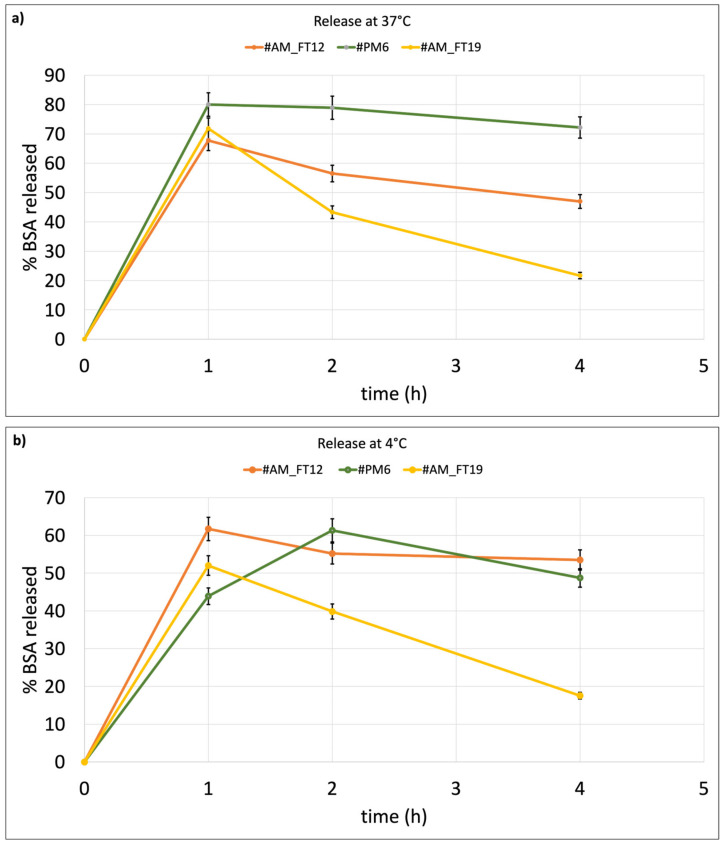
BSA in vitro release from liposome batches AM_FT12, PM6, and AM_FT19: (**a**) at 37 °C and (**b**) at 4 °C.

As mentioned in the Section 4.2., BSA in vitro release tests were carried out on those liposomes showing higher EE% and keeping their size below 200 nm. The selected batches were AM_FT19 (cationic liposomes, EE% = 6 ± 0.8%), PM6 (cationic liposomes, EE% = 4.57 ± 0.2%) produced by microfluidic and passive-loading method, and #AM_FT12 (neutral liposomes, EE% = 5.5 ± 0.3%). Thus, the in vitro release test was carried out on two liposome batches prepared by microfluidics and loaded with BSA using FT as the active-loading method, and one batch of liposomes prepared by microfluidics was also used as the passive-loading method. One of the two liposome batches loaded by the FT active-loading method contained cationic lipids (AM_FT19), and the other one was made from neutral lipids (AM_FT12). The liposome batch loaded with BSA by the passive method (PM6) was made from cationic lipids. All tested liposome batches showed burst release after 1 h incubation at 37 °C, but PM6 showed the highest, reaching 80% BSA release in 1 h, and AM_FT12 the lowest (60%) (Figure 3a) at 4 °C (Figure 3b). Neutral liposomes loaded with the active method (AM_FT12) show an initial BSA burst release both at 37 °C (68 ± 6.6%) and 4 °C (62 ± 5.7%), which, however, is followed by a more gradual release over time, with both conditions not reaching 100% release in 4 h.

Finally, the cationic liposomes loaded by the passive method (PM6) show 80 ± 7.2% BSA burst release at 37 °C (followed by a plateau up to 4 h in vitro release test. Burst release of BSA from the same batch PM6 at 4 °C (Figure 3b) is 44 ± 5.3%, i.e., significantly lower if compared to the same batch tested at 37 °C, and it is significantly lower for all test times. The results demonstrate better time control release of BSA from passively loaded cationic liposomes (batch PM6) compared to actively loaded cationic liposomes (batch AM_FT19). This could be explained by the fact that BSA, encapsulated during the concomitant formation of liposomes, manages to remain better encapsulated within the liposome core and less absorbed on the liposome charged surface. Moreover, when BSA is encapsulated into cationic liposomes by both passive and active methods (batches PM6 and AM_FT19), the temperature of the release medium (4 °C or 37 °C) becomes a discriminant variable because of the more rigid structure of liposomes at 4 °C, which better retains the BSA encapsulated in their core.

To confirm the release delay, after 4 h in vitro release test, the liposomes were recovered by centrifugation and the pellet treated with TritonX 2% *v*/*v* in PBS for quantification of the remaining encapsulated protein. Quantification confirmed that the missing amount of BSA was still embedded into the liposomes. The residual BSA% founded in liposomes after release test was 22.4 ± 6.18% for batch PM6, 43.4 ± 2.5% for batch AM_FT12, and 37.05 ± 9.18 for AM_FT19 after incubation at 37 °C and 54.2 ± 3.4% for PM6, 33.6 ± 4.9% for AM_FT12, and 47.29 ± 5.4 for AM_FT19 after incubation at 4 °C, respectively.

### 2.4. Biological Characterization

#### 2.4.1. Cell Viability 

**Figure 4:**
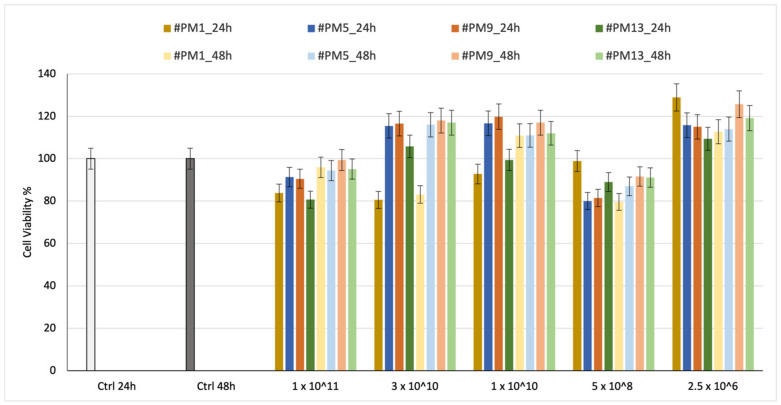
Cell viability % results of MTT assay performed on HNDF at 24 h and 48 h using neutral and charged DSPC:Chol-based empty liposomes.

Cell viability was preliminarily evaluated on HNDF for 24 h and 48 h. The % viability results are shown in Figure 4.

The tests were carried out on empty liposomes because BSA is well known to be cytocompatible and is sometimes also used in cell culture media; therefore, it was preferred to concentrate on the effect of the liposome alone. 

Cell viability was reported in relation to the number of liposomes placed in contact with HNDF cells.

The results of cell viability after incubation with 1 × 10^11^ liposomes, which is the highest liposome concentration, are between 80% and 100%, demonstrating the liposomes’ biocompatibility. No significant difference is highlighted between neutral and charged liposomes. In all cases, the viability after 24 and 48 h is confirmed with values higher than 80%, indicating suitable cytocompatibility.

#### 2.4.2. In Vitro Cellular Uptake 

The in vitro cellular uptake test was performed on surface-charged empty liposomes of the same compositions as explained above but supplemented with a Fluorescein-DHPE fluorescent marker. The liposome batches #PM5-fluo, #PM9-fluo, and #PM13-fluo were produced by the same microfluidics protocol set up for empty surface-charged liposomes, and they were incubated with cells at 1 × 10^10^ concentration. This liposome concentration was selected because it yielded the best vitality values in all cases in 24 h and 48 h with HNDF.

**Figure 5:**
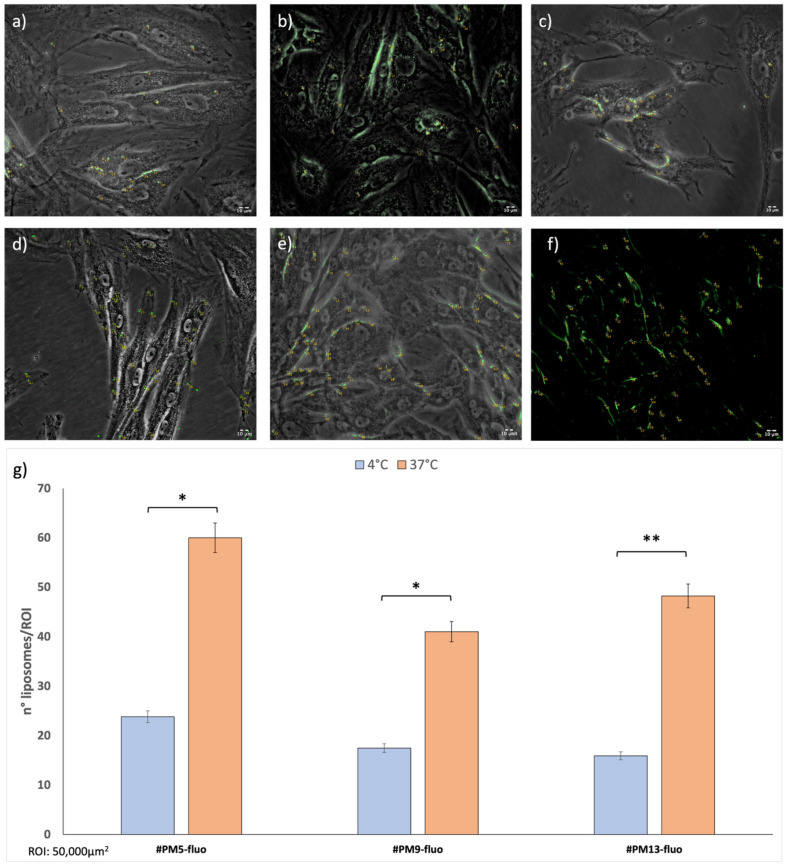
(**a**–**f**) Fluorescent microscope images of fluorescent liposomes (**a**) and (**d**) PM5-fluo, (**b**) and (**e**) PM9-fluo and (**c**,**f**) PM13-fluo incubated with HNDF. Images were taken at 20× magnification. (**g**) number of uptaken liposomes counted with ImageJ software. A *p*-value less than 0.05 (* *p* < 0.05) was considered statistically significant; a *p*-value higher than 0.05 (** *p* > 0.05) was considered not statistically significant.

Uptake was evaluated after 1 h, and liposomes were quantified using ImageJ software (Figure 5).

The number of liposomes counted for each sample was normalized for region of interest (ROI = 50,000 μm^2^). Uptake at 4 °C was always significantly lower compared to the same timing point at 37 °C. Cationic liposomes (batch #PM5-fluo) showed greater results in terms of uptake both at 37 °C and 4 °C compared to the other liposomes tested (batches #PM9-fluo and #PM13-fluo).

Moreover, it is evident how the positively charged liposomes enter the cell retaining a spherical shape, and only a few cases of aggregation were highlighted. On the contrary, zwitterionic and anionic liposomes show more tendency to interact with cells through the mechanism of fusion with the cell membrane, making the cell walls sensitive to the fluorescence of the lipid DHPE integrated into the structure.

Cellular uptake after 4 h shows that both at 4 °C and at 37 °C, there is a majority of positively charged liposomes (#PM5-fluo) inside the HNDF cells (Supplementary Data Figure S2). This result is compatible with the fact that cells have a tendency toward a negative surface charge and, therefore, by electrostatic interaction, there is an attraction between opposite charges [33].

## 3. Discussion

On the basis of the results obtained, the discussion wants to point out those aspects related to encapsulation methods and liposome composition, such as: their influence on encapsulation efficiency and on in vitro release behavior of BSA by keeping the liposome size below 200 nm and almost monodisperse size distribution (PDI below 0.7). Eventually, a brief mention is also addressed to potential industrial application of the preparation methods experimented. As far as the encapsulation of protein into liposomes is concerned, Hwang et al. report that the efficiency of liposomal encapsulation of proteins is generally low and is affected by: (i) phospholipid concentration, (ii) buffer pH, (iii) protein concentration, (iv) liposomes size, (v) protein dimension (Mw), and (vi) electrostatic interactions. The authors tested the encapsulation of enzymes with different MW into neutral liposomes made of DPPC (1,2-dipalmitoyl-sn-glycero-3-phosphocholine) and concluded that encapsulation efficiency depended mainly on liposome size [34]. In our work, we wanted to keep the liposome size constant below 200 nm and protein MW. Moreover, the protein MW is not a variable in our work because only BSA was experimented with. The liposome encapsulation methods and the lipid composition were the variables tested in our work toward BSA encapsulation efficiency. Indeed, the characteristics of the liposome lipid components, such as their surface charge, can act as a retention mechanism for molecule loading and transport.

As far as BSA encapsulation into liposomes is concerned, even if several studies can be found in the literature that evaluate how to improve the encapsulation efficiency of BSA into liposomes, none of them fit with our assumptions or compare passive and active methods to load proteins into liposomes.

For example, Liu et al. studied a method to increase BSA loading into soybean phospholipid:cholesterol:Tween-80 liposome and obtained 33.6 ± 9.1% EE%. However, the resulting liposomes had dimensions exceeding 1000 nm, and, therefore, they are out of our scope because they are not suitable for parenteral administration or organ perfusion [35]. Okamoto et al. tested different BSA concentrations and lipid molar ratios to increase EE% in egg PC:cholesterol:DSPE-PEG2000 liposomes. By increasing protein concentration (200 mg/mL) and lipid molar ratio (12:6:1), greater BSA content was achieved (2.64 ± 0.18 mg/mL) [36]. However, the limit of this work is that the amount of protein to be used for the preparation is high, and it is not possible to translate it for all proteins. In our work, 10 mg/mL BSA (0.75 mL) aqueous solution was used in order to limit protein consumption. Using the microfluidic technique as the passive-loading method, Weaver et al. [37] produced liposomes composed of DSPC, DMPC, DPPC, or DOPC with cholesterol (2:1 ratio, respectively, at 1 mg/mL). Increasing BSA concentrations in PBS were tested (0.5–4 mg/mL). The results obtained confirmed that the microfluidic technique, compared to the traditional thin layer method, allows for obtaining liposomes with more controllable dimensions (nm and PDI) and with an improvement in BSA loading. We are aware that microfluidics is a superior method of manufacturing liposomes and of loading them, at least by the passive method. For this reason, we chose this method to prepare empty liposomes.

However, scarce literature has been found that evaluates the active and passive methods of liposome drug loading, and it refers to drug molecules different from proteins [38,39].

Therefore, we wanted to put a step forward, investigating various methods of active loading a model protein inside preformed liposomes obtained by the microfluidic technique. The rationale is to evaluate which method achieves a better overall EE% and also overcomes the limitations associated with the traditional passive delivery method, such as low encapsulation efficiency, non-encapsulated drug loss, and organic solvents residual in the batch [17]. From the data obtained, it emerged that the microfluidic passive-loading method resulted in an EE% (about 4.0) 20% higher with respect to the microfluidic and SC active-loading methods. Instead, FT (AM_FT12, 5.5 ± 0.3 EE%) and EP (AM_EP16, 8.2 ± 0.9 EE%, AM_EP17, 7 ± 1 EE%, and AM_EP18, 5.3 ± 0.6 EE%, respectively) active-loading methods allowed for doubling up BSA EE%. Unfortunately, EP also caused a dramatic increase in liposome size (>600 nm), which is not suitable for the purpose of the parenteral administration route. The results also demonstrated that the addition of 5%mol of DOTAP (cationic lipid) in combination with BSA (negatively charged at pH 7.4) permitted a 13% EE% increase by the passive-loading method (PM6, 4.57 ± 0.2 EE%) and a 45% EE% increase by the FT active-loading method (AM_FT19, 7.2 ± 0.8) while keeping liposomal dimensions in a range suitable for injection (<200 nm). These results confirm that FT is suitable as an active-loading method and that lipid composition significantly affects protein-loading ability. The microfluidic and electroporation active-loading methods resulted in affected liposome size. As far as microfluidics is concerned, the active-loading mechanism is based on BSA diffusion rather than lipid layer perturbation, and this might cause liposome swelling. Instead, the increase in liposome size due to electroporation might be due to its high perturbating effect on the lipid layer.

The laboratory scales tested in this work show the active protein principle that each method intrinsically spent was the same, and a direct comparison can be performed among the BSA-loading methods tested in terms of EE%. Therefore, the higher the EE%, the lower the amount of active principle lost during the process, the higher the process efficiency. All the methods tested have been carried out at the lab scale, and their transfer to the industrial scale may require even a change in the type of apparatus. For example, freezing in liquid nitrogen at the industrial scale could be very different from the same process performed at the lab scale, and it could require shorter times. This is undoubtedly a limitation of this work, whereas the microfluidic method is the only one that can be easily transferred to industrial applications because it is based on automated apparatuses that are already on the market at the lab and industrial scales. Undoubtedly, the microfluidic technique is confirmed to be a valid method for manufacturing protein-loaded liposomes both by the passive method and as a platform for actively increasing the protein EE%. Moreover, the speed of the technique (few minutes) and its reproducibility as long as liposome morphological properties are concerned (size and PDI) offer the possibility of using the MF as a platform for the loading of proteins.

As far as the functional properties of the BSA-loaded liposomes are concerned, the protein release rate from neutral liposomes seems not to be affected by incubation temperature both in active- and passive-loaded liposomes. The result is in keeping with what was reported in the literature by Weaver et al. [37].

In contrast, BSA release from cationic liposomes, both by passive and active methods, is affected by incubation temperature and significantly slowed down at 4 °C. 

BSA has intrinsic properties to interact with lipids and, as a consequence of it, perturbing liposome membrane structure. Thus, many of the differences shown in terms of BSA content and release from the different liposomes are likely caused by the interaction of BSA with liposome membranes and their perturbation. Therefore, the BSA release profile upon different experimental conditions has also been likely to be influenced by the intrinsic ability of the protein to adsorb and bind to phospholipid membrane surfaces, and this phenomenon is particularly evident when cationic lipids and active protein-loading methods are used. It is clear that important differences in terms of encapsulation efficiency by the different methods may arise from the different nature (size, charge, lipid affinity, and conformation) of the protein of interest. Taking into account BSA properties, it can be stated that the passive-loading method leads to better control of the BSA in vitro release rate. The term in vitro is important to be underlined because, in an in vivo experiment, interaction with plasma proteins will have to be also considered. 

Another important step in the discussion is added by the results of cellular uptakes carried out at 37 °C and 4 °C in 4 h (Figure 5). They show how cationic liposomes are able to be greatly internalized already after 1 h of incubation with the cells. Thus, it can be stated that even the protein release rate from cationic liposomes actively loaded with a protein whose IP was <7.4 might be compatible with protein-loaded liposome supplementation in organ perfusion protocols (temperature 4 °C and times 1–4 h).

Interestingly, all charged liposomes were cytocompatibile at the tested concentration, independently of their surface charge.

## 4. Materials and Methods

### 4.1. Materials

1,2-distearoyl-sn-glycero-3-phosphocholine (DSPC, MW = 790.145 g/mol), 1,2-dioleoyl-3-trimethylammonium-propane (DOTAP, MW = 698.54 g/mol), 1,2-dioleoyl-sn-glycero-3-phospho-L-serine (DOPS, MW = 810.025 g/mol), and 1,2-dipalimitoyl-sn-glycero-3-phosphoethanolamine (PE, MW = 691.95 g/mol) were purchased from Avanti-Polar Lipids Inc. (Alabaster, AL, USA). Cholesterol (Chol, MW = 386.65 g/mol), bovine serum albumin (BSA) 69.324 MW, IP 4.5, phosphate buffer (PBS), and Fluorescein-DHPE, (N-(Fluorescein-5-thiocarbamoyl)-1,2-dihexadecanoyl-sn-glycero-3-phosphoethanolamine, Triethylammonium Salt-MW = 1182.54 g/mol) were purchased from Sigma Aldrich (Milan, Italy). BCA protein assay kit and MTT (3-(4,5-Dimethylthiazol-2-yl)-2,5-Diphenyltetrazolium Bromide) (ThermoFisher Scientific, Rockford, IL, USA) were used. Normal human dermal fibroblasts (NHDFs) were purchased from ATCC (American Type Culture Collection, Manassas, VA, USA). Growing medium Dulbecco Modified Eagle’s Medium (DMEM) was purchased from Microgem Laboratory Research (Milan, Italy). Where not specified, all reagents and solvents used are of analytical purity.

### 4.2. Methods

**Figure 6:**
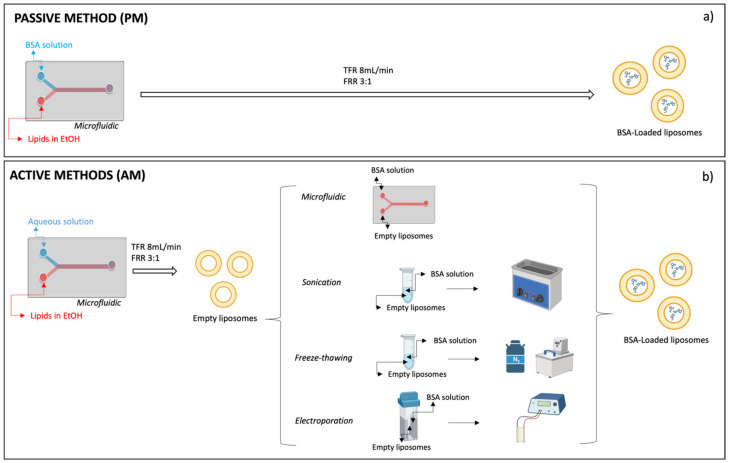
BSA-loaded liposomes preparation by passive method (**a**) and active methods (**b**).

The procedures for the preparation of BSA-loaded liposomes by the passive method (PM) and active method (AM) are schematized in Figure 6.

#### 4.2.1. Empty Liposome Preparation by Microfluidic Technique

The empty DSPC:Chol liposomes were prepared by the microfluidic technique with a Nanoassemblr Benchtop apparatus (Precision Nanosystems Inc., Vancouver, BC, Canada) equipped with a staggered herringbone micromixer (SHM). The micromixer cartridge dimensions were 6.6 × 5.5 × 0.8 cm (w × d × h), and it was made of polypropylene, viton, and cyclic olefin copolymer. The cartridge’s mixing channel was 200 × 79 μm (w × h), and the herringbone structure was 31 μm high and 50 μm thick. There was an angle of 45° between the ridges and the long axis of the channel. The microfluidic device consisted of a Y-junction, known as a staggered herringbone, followed by a staggered mixing region. The staggered herringbone structures induce rapid mixing by chaotic advection [23].

In this work, channel 1 of the cartridge was loaded with aqueous-based solutions while a lipid composition of DSPC:Chol 50:50 molar ratio in ethanol solution (10 mM) was loaded in channel 2. Both inlet streams were controlled by syringe pumps connected to a computer that controlled the whole process. The total flow rate (TFR) of aqueous and ethanolic streams was 8 mL/min, and the flow rate ratio (FRR) was 3:1, as set in a previous work of the same authors and reported in Table 3—#PM1 [40]. The liposomes preparation process with Nanoassemblr was performed at room temperature (25 ± 3 °C).

#### 4.2.2. BSA-Loaded Liposomes Preparation by Passive Method (PM) and Active Method (AM)

BSA was used as a model molecule that simulates protein drug molecules. The BSA molecular mass was 66 kD, the isoelectric point (IP) = 4.5, and the overall molecule size was 7.1 nm [41]. BSA was loaded into liposomes using the passive method, contextual to liposome preparation (#PM2), as the control. The microfluidic process conditions were those already reported above in Table 3 [40]. BSA was solubilized in MilliQ water (brought to pH 7.4 by NaOH addition) supplemented with trehalose 20% *w*/*w*. MilliQ water was used in order to avoid BSA dimerization, as happens in PBS buffer solution, and to keep its original MW 66 KDa [42]. Trehalose 20% *w*/*w* was added to improve the amount of loaded BSA and stabilize BSA structure, as reported in the literature [43,44].

BSA-loaded liposomes were prepared by the passive method through the microfluidic technique. The experimental setup was as reported for the preparation of empty liposomes (see Section 4.2.1.), but channel 1 of the microfluidic device was loaded with 10 mg/mL BSA aqueous solution (0.75 mL), brought to pH 7.4 by NaOH addition and supplemented with trehalose 20% *w*/*w*.

BSA was encapsulated with the active method using preformed empty liposomes (#PM1) obtained by the microfluidic technique as reported above.

Microfluidic, sonication, freeze–thawing, and electroporation were the four tested active encapsulation methods and were performed as follows.

- Microfluidic (MF). Nanoassemblr benchtop apparatus was used as described above. A total of 10 mg/mL BSA aqueous solution (pH 7.4) supplemented with 20% *w*/*w* of trehalose and preformed liposomes DSPC:Chol 50:50 suspension in water were loaded into the microfluidic cartridge through inlet stream channel 1 and inlet stream channel 2, respectively. The FRR between the aqueous solution containing BSA and liposome suspension was maintained at 3:1 (0.75 mL BSA solution:0.25 mL liposome suspension). The batches obtained were centrifuged at 16,400 rpm, 4 °C, for 30 min to separate the BSA-loaded liposomes from the non-encapsulated BSA. Table 4 shows the batches prepared with this technique by varying the total flow rate from 4 mL/min to 12 mL/min (from #AM1 to #AM6).
- Sonication (SC). Empty DSPC:Chol 50:50 liposomes pellets were suspended in 0.75 mL aqueous solution (pH 7.4) containing BSA (10 mg/mL) and trehalose (20% *w*/*w*) or in PBS buffer (pH 7.4). Sonication was performed with a SONICA^®^ ultrasonic cleaner Soltec^®^ (Milan, Italy), working with a frequency of 37 kHz. The sonication cycles were as follows. The liposomes–BSA mixture was cooled in an ice bath for 5 min and sonicated for 24 s (6 cycles of 4 s pulse and 2 s pause) or 60 s (10 cycles of 4 pulse and 2 pause) [43]. After a 2 min cooling in an ice bath, the batches were re-sonicated under identical conditions. The sonicated batches were centrifuged at 16,400 rpm at 4 °C for 30 min to separate the BSA-loaded liposomes from the non-encapsulated BSA. Table 4 summarizes the sonication conditions tested and the batches prepared with this technique (from #AM7 to #AM9)
- Freeze–thawing method (FT). DSPC:Chol 50:50 liposomes pellets (#PM1) were suspended in 0.75 mL aqueous solution (pH 7.4) containing BSA (10 mg/mL) and trehalose (20% *w*/*w*) or in PBS buffer (pH 7.4). The liposomes–BSA mixture was subjected to three or five cycles of freezing in liquid nitrogen (−196 °C) and thawing in a water bath at 37 °C. After the freeze–thawing cycles (3 or 5), the batches were centrifuged at 16,400 rpm, 4 °C, for 30 min to separate the BSA-loaded liposomes from non-encapsulated BSA. Table 4 reports the batches prepared with this technique and the related process conditions (from #AM10 to #AM12)
- Electroporation (EP). DSPC:Chol 50:50 liposome pellets were resuspended in 0.75 mL aqueous solution (pH 7.4) containing BSA (10 mg/mL) and trehalose (20% *w*/*w*) or in PBS buffer (pH 7.4). Electroporator Gendrive IGEA (MU-GENEDRIVE_EN-Rev. 2.0 June 2021 Software Distribution 1.5) equipped with disposable UV-grade methacrylate cuvette (2 mm plate distance) was used. Suspensions were electroporated for a single cycle (n° pulse = 8, length = 10 μs) at increasing voltage rates (100 V, 200 V, and 300 V). After electroporation, the batches were centrifuged at 16,400 rpm, 4 °C, for 30 min to separate the BSA-loaded liposomes from the non-encapsulated BSA. Table 4 reports the batches prepared with this technique (from #AM13 to #AM18).

#### 4.2.3. Charged Liposomes Formulations

Liposome formulation was implemented using cationic, anionic, or zwitterionic lipids to increase BSA EE%. As the cationic lipid, Dioleoyl-3-trimethylammonium propane (DOTAP) was selected, as having a quaternary amine, it is always positive at any pH of the medium. Starting from lipid composition DSPC:Chol 50:50, DOTAP was added until it reached a 5% molar ratio. Cationic lipid-based vectors have been shown to perform extremely efficiently in cellular transfection in vitro [45]. 1,2-dioleoyl-sn-glycero- 3-phospho-L-serine (DOPS) anionic lipid was added to DSPC:Chol 50:50 composition until it reached a 5% molar ratio. Negatively charged lipids were selected to create liposomes with a surface charge able to mimic biological extracellular vesicles [46]. Lipid phosphatidylethanolamine (PE) was selected as a zwitterionic component, able to change its surface charge depending on the pH environment. PE was added at increasing concentration until it reached a 5% molar ratio. A linear relation between PE and pH environment was performed to evaluate liposome surface charge changes.

The empty and BSA-loaded charged liposomes were produced through the microfluidic technique as the passive method and freeze–thawing technique as the active method, as explained below for batch #AM_FT12. 

Liposome production using the microfluidic technique was performed following the optimized parameters: 8 mL/min (TFR) and 3:1 (FRR). BSA (10 mg/mL) was solubilized in MilliQ water (pH = 7.4) supplemented with trehalose (20% *w*/*w*).

Empty liposomes obtained through the microfluidic technique were used to perform BSA encapsulation with the active method. The pellets were resuspended in 1 mL of BSA solution (10 mg/mL) in water containing trehalose (20% *w*/*w*). The liposomes–BSA suspensions underwent five cycles of freezing in liquid nitrogen and thawing in a water bath at 37 °C. At the end of the freeze–thawing cycles, the liposomes were centrifuged at 16,400 rpm, 4 °C, for 30 min to separate free BSA from liposomes loaded with BSA.

Table 5 reports the lipid composition of charged liposomes (molar ratio), either empty or BSA loaded through passive and active methods.

### 4.3. Liposomes Characterization

The physicochemical properties of nanomaterials influence their behavior in the biological environment [47]; for this reason, liposomes were characterized for their dimensions, surface charge, and batch yield in terms of particles/mL. Liposomes loaded with BSA were also characterized for their encapsulation efficiency (EE%) and drug content (DC%). Morphological characterization with TEM was performed on empty charged liposomes.

#### 4.3.1. BSA-Loading Determination

BSA loaded inside liposomes was extracted, collecting the pellets through centrifugation (16,000 rpm, 4 °C, 30 min) and then resuspending them with TritonX 2% *v*/*v* in PBS aqueous solution. The suspension was vortexed for 1 min (Advanced Vortex Mixer Zx3 Velp scientifica^®^, Milano, Italy) and sonicated for 1 min to completely disrupt liposomes and extract BSA. The amount of BSA extracted from liposomes was quantified by a BCA kit and UV-VIS spectrophotometry at 562 nm with a Microplate Photometer MPP-96 bioSan^®^ [48]. A BSA calibration curve was prepared in the BSA concentration range between 0.025 mg/mL and 2 mg/mL (R^2^ = 0.9967; y = 1.091x + 0.028).

The EE% of BSA was calculated with the following equation (Equation (1)).

(1) $${\mathrm{EE}}_{\mathrm{direct}}\%=(\mathrm{Weight}\;\mathrm{of}\;\mathrm{protein}\;\mathrm{in}\;\mathrm{liposomes}/\mathrm{Weight}\;\mathrm{of}\;\mathrm{feeding}\;\mathrm{protein})\;\times \;100$$

DC% of BSA was calculated with the following equation (Equation (2)).

DC% = (Weight of protein in liposomes/Weight of liposomes) × 100(2)

The weight of liposome batches was evaluated on liposome batches that underwent freeze-drying protocol at −25 °C for 8 h and then freeze-dried for 24 h (−50 °C, 0.02 mBar) (Lio-5Pascal, Milano, Italy).

The active method that led to liposomes with the highest BSA EE% while keeping the liposome size lower than 200 nm with a PDI < 0.3 [31] was selected for further analysis, implementing the lipid composition using charged lipids, performing characterization and in vitro release test at 4 °C and 37 °C.

#### 4.3.2. BSA In Vitro Release Test

In vitro release tests were performed on liposomes that showed greater BSA EE%, maintaining a suitable dimension lower than 200 nm, and obtained through the passive and active methods.

The tests were performed at 37 °C and 4 °C in PBS (pH7.4). The temperature simulating human body physiologic temperature was 37 °C, while 4 °C was selected to simulate hypothermic machine perfusion temperature (4–12 °C for 4 h) with the aim of also testing the liposomes for those potential applications where a drug supplement could be required along isolated and explanted organ perfusion protocols [49]. BSA-loaded liposome pellets were recovered through centrifugation at 16,400 rpm, 30 min, 4 °C and resuspended in 1 mL of PBS buffer solution (pH = 7.4). At defined times (1 h, 2 h, and 4 h), the samples were centrifuged to precipitate liposomes and recover the released BSA from 100 µL supernatant. An equal volume of fresh PBS buffer (100 µL) always added to repristinate the initial dissolution volume and keep sink conditions. A Pierce^®^ BCA protein assay kit was used to quantify the BSA released, as explained above. The amount of BSA released was determined using the following formula (Equation (3)):

(3) $$Drug\;release\;\left(\%\right)=\frac{R_{t}}{L}\times 100$$

where *L* represents the amount of drug loaded into liposomes and *R_t_* is the cumulative amount of drug released at time *t*.

#### 4.3.3. Particle Size and Zeta Potential Determination

Dynamic light scattering (DLS) and nanoparticle tracking analysis (NTA) are two orthogonal methods for measuring particle size in a sample. Both technologies use the theory of Brownian motion by analyzing random changes in the intensity of light scattered by particles in solution, but compared to DLS, NTA has a higher resolution and also allows for themeasurement of particle concentration in terms of particles/mL [50].

- Dynamic Light Scattering analysis (DLS)

Nicomp 380ZLS (Particle Sizing Systems, CA, USA) was used for the determination of liposome size. The instrument uses a dynamic light scattering (DLS) method and z-potential. The main parameters set up for particle size analysis were: channel 10, intensity 100 kHz, temperature 25 °C, viscosity 0.933, and liquid index of refraction 1.333.

Dimensional analysis was performed on pure samples, while for the z-potential analysis, 100 μL of liposome suspension was placed in 2 mL of NaCl solution (0.01 M, pH = 7.4). Analyses were performed at 10 mV electric field intensity and at room temperature (25 ± 2 °C).

- Nanoparticle tracking analysis (NTA)

Nanoparticle tracking analysis (NTA) was performed using NanoSight NS300 Malvern Panalytical Ltd (Malvern, UK) for size characterization and quantification of liposomes. The liposome suspensions were loaded into a camera, which was subsequently illuminated by laser light (635 nm). The particles in the sample scattered the light beam, which was detected by the 20× magnification. The microscope was connected to a camera that captured video (30 frames per second) of the liposomes. The samples were diluted in sterile deionized water (dilution factor: 5 × 10^2^) for the analysis and injected into a sample chamber using an injection pump (3.0 μL/min). Analyses were performed at room temperature (25 ± 2 °C).

#### 4.3.4. Transmission Electron Microscopy (TEM)

Liposome shape was analyzed with a JEOL JEM-1200EXIII electron microscope equipped with a TEM Mega View III CCD camera. For TEM analysis, 100 μL of the sample was adsorbed on a carbon film grid and was diluted with 900 μL of PBS. Then, a drop of uranyl acetate was added. TEM images were acquired with magnifications of 150 K and 200 K. Each batch was analyzed in triplicate. The images performed in triplicate for each batch were then processed with ImageJ software, which allows you to adjust brightness, contrast, and sharpness. NiBlack thresholding was used to obtain local threshold images. Considering the perfect spherical shape of liposomes, we used the “polygon shape” tool to define the perimeter of the liposome and calculate the Ferret’s diameter. The program was then able to supply the diameter and circularity (where values close to 1 indicate a perfectly spherical shape while values close to 0 indicate a straight line) of the traced polygon. Measurements were performed in triplicate for all samples.

### 4.4. Biological Characterization

#### 4.4.1. Cell Viability

Liposome biological characterization was performed by MTT (3-(4,5-Dimethylthiazol-2-yl)-2,5-Diphenyltetrazolium Bromide) assay protocol for cell viability. MTT assay was performed for neutral and charged empty liposomes with normal human dermal fibroblast cells (NHDF, P.6).

For the MTT assay, in each well of a 96-well plate, 30,000 cells were sown with 200 μL of culture medium (DMEM, 10% FBS, 1% penicillin, and streptomycin). Then, the multi-wells were incubated for 24 h at 37 °C and 5% CO_2_ to guarantee cell adhesion. After 24 h, both well plates were treated with increasing concentrations of liposomes (particles/mL) calculated from the NTA process yield (10^11^ particles/mL).

Then, well plates were incubated for 24 h and 48 h. After the incubation period, the medium was removed, and the cells were washed twice with PBS (pH 7.4). A total of 10 μL of the MTT labeling reagent (5 mg/mL in PBS) was added to each well and filled with another 190 μL of fresh PBS. Plates were incubated for 2 h and 30 min in a humidified atmosphere (37 °C, 5% CO_2_). Subsequently, MTT was removed, and 100 μL of DMSO was added to each well to dissolve formazan crystals. Finally, the plate, after being kept under shaking for 45 min, was read under a microplate photo-reader (570 nm). MTT assay was performed in triplicate, and the data were reported as average ± SD. Statistical analyses were performed using Microsoft Excel (Office 365 Microsoft, Redmond, WA, USA). A *p*-value less than 0.05 (* *p* < 0.05) was considered statistically significant.

#### 4.4.2. In Vitro Cell Uptake

For the evaluation of cellular uptake, empty liposomes with different surface charges were produced with Fluorescein-DHPE for fluorescent uptake detection.

Fluorescein-DHPE (1 mg/mL in EtOH) was used as a fluorescent marker to mark the lipid layer of liposomes (1% molar ratio). The same preparation protocol setup for empty liposomes was used through the microfluidic technique.

The uptake of fluorescent liposomes by human normal dermal fibroblas (HNDFs) was evaluated with a Leica DM IL LED with an ebq 50 ac-L (©Leica Microsystems) fluorescence microscope. The fluorescent liposome concentration was 10,000,000,000 liposomes/mL.

A total of 600,000 cells were seeded into 35 mm glass-bottom Petri dishes in 2 mL of medium (DMEM, 10% fetal bovine serum FBS, 1% penicillin, and streptomycin). The dishes were incubated (37 °C, 5% CO_2_) for 24 h to guarantee cell adhesion. Subsequently, the medium was replaced with 700 μL of fresh growth medium in each dish, and 300 μL of fluorescent liposome suspension was added.

Cell uptake of all the fluorescent liposome samples (PM5-fluo, #PM9-fluo, and #PM13-fluo) was observed after 1, 2, and 4 h of incubation at 37 °C and 4 °C. At these tie points, the Petri dishes were washed with PBS to remove non-uptaken liposomes and visualized with a fluorescent microscope to evaluate liposome cellular uptake. Quantification was performed by processing microscope images through ImageJ software.

### 4.5. Statistical Analysis

All experiments were carried out in triplicate (n = 3) unless otherwise stated. All data are presented as mean ± standard deviation (n = 3 unless stated otherwise). MicrosoftTM Excel was used to plot all graphs. A statistical analysis tool was performed in Microsoft Excel (Office 365 Microsoft, Redmond, WA, USA) using analysis of variance (ANOVA). A *p*-value less than 0.05 (*p* < 0.05) was considered statistically significant, 0.05 < ** *p* ≤ 0.1 was marginally statistically significant, and a *p*-value higher than 0.1 (*p* > 0.1) was not statistically significant and indicated strong evidence for the null hypothesis.

## 5. Conclusions

This preliminary study demonstrated how the encapsulation method and electrostatic protein–lipid interaction play a decisive role in defining BSA EE% and liposome size. These parameters, therefore, also influence the release of the protein from the liposomal system in vitro. The results obtained demonstrated the FT active-loading method to be the more convenient one to load BSA. The results showed that EP also showed promising results, and it will be more thoroughly investigated with other types of lipid compositions and proteins. Eventually, EP, already used for gene transfer, could be exploited for the improvement in the loading capacity of nanosystems and for innovative therapeutic strategies [51].

## Figures and Tables

**Table 1:** ImageJ characterization of charged liposomes.

Batch	Circularity	Diameter (nm ± SD) from ImageJ Elaboration
PM5	0.93 ± 0.004	125.5 ± 11
PM9	0.89 ± 0.03	112.8 ± 7.2
PM13	0.91 ± 0.01	133.8 ± 24
AM_FT12	0.88 ± 0.05	115.3 ± 12
AM_FT19	0.90 ± 0.03	166.1 ± 19

**Table 2:** Charged liposomes size, Z-potential, EE%, and yield of production.

Batch	Dimension	PDI	Z-Potential (mV)	EE%	DC%	Process Yield (Particles/mL)
PM3	118.5 ± 39.8	0.210 ± 0.02	+3.4 ± 1.7	-	-	-
PM4	180 ± 12.1	0.230 ± 0.03	+1.3 ± 0.6	1.99 ± 0.1	7.09 ± 0.4	1.55 × 10^11^ ± 2.88 × 10^10^
PM5	160.1 ± 6.2	0.102 ± 0.01	+20 ± 3.0	-	-	1.42 × 10^11^ ± 1.38 × 10^10^
PM6	131.2 ±11.4	0.140 ± 0.01	+18 ± 1.3	5.57 ± 0.2	19.89 ± 1.7	1.23 × 10^11^ ± 6.69 × 10^9^
AM_FT19	165.8 ± 5.9	0.268 ±0.02	+16 ± 3.1	7.2 ± 0.8	25.71 ± 1.1	1.41 × 10^11^ ± 1.54 × 10^10^
PM7	140 ± 6.1	0.443 ± 0.05	0 ± 0.7	-	-	-
PM8	163.3 ± 15.5	0.434 ± 0.07	+2.6 ± 0.7	1.80 ± 0.2	6.43 ± 0.9	1.33 × 10^11^ ± 3.37 × 10^10^
PM9	131.8 ± 70.3	0.530 ± 0.04	−9.9 ± 1.2	-	-	1.56 × 10^11^ ± 7.68 × 10^9^
PM10	132.2 ± 8.8	0.432 ± 0.02	−11.2 ± 1.1	2.25 ± 0.2	8.04 ± 1.4	1.25 × 10^11^ ± 4.12 × 10^10^
AM_FT20	144.7 ± 15.3	0.501 ± 0.05	−12.2 ± 0.9	1.9 ± 1.1	6.78 ± 1.1	1.33 × 10^11^ ± 5.18 × 10^9^
PM11	140 ± 17.7	0.277 ± 0.06	−13.4 ± 2.2	-	-	-
PM12	151 ± 21.1	0.282 ± 0.05	−14.5 ± 3.2	2.28 ± 0.4	8.14 ± 0.8	1.22 × 10^11^ ± 1.18 × 10^10^
PM13	163.8 ± 44.1	0.121 ± 0.02	−39.3 ± 2.1	-	-	1.69 × 10^11^ ± 9.08 × 10^0^
PM14	157.7 ± 20	0.219 ± 0.02	−41.5 ± 2.7	3.1 ± 0.1	11.07 ± 0.6	1.11 × 10^11^ ± 4.08 × 10^10^
AM_FT21	170 ± 10.5	0.301 ± 0.03	−40.7 ± 1.8	1.7 ± 0.1	6.07 ± 0.8	1.16 × 10^11^ ± 9.66 × 10^9^

AM: active method; FT: freeze and thawing; DC%: drug content; EE%: encapsulation efficiency; PDI: polydispersity index; PM: passive method.

**Table 3:** Empty and BSA-loaded liposomes (DSPC:Chol 50:50) obtained using passive method through microfluidic technique [40].

	#PM1	#PM2
DSPC:Chol	50:50	50:50
TFR (mL/min)	8	8
FRR	3:1	3:1
Trehalose *w*/*w %*	20%	20%
BSA (mg/mL)	-	10
MilliQ water (pH 7.4)	yes	yes
Dimension (nm)	122.9 ± 0.7	139.8 ± 5.4
PDI	0.23	0.28
Z-pot (mV)	+1.1 ± 0.7	−2.4 ± 0.5
EE%	-	4.0 ± 1.3%
Yield of process (particles/mL)	3.08 × 10^11^ ± 2.52 × 10^10^	3.02 × 10^11^ ± 3.22 × 10^10^
Yield of process (mg)	2.31 ± 0.8 mg	2.37 ± 0.4 mg

**Table 4:** Process parameters used to prepare BSA-loaded liposomes with each active method.

Batch	BSA (10 mg/mL) Trehalose (20% *w*/*w*)	
Microfluidic Method (MF)		Total Flow Rate (mL/min)
AM_MF1	PBS	4
AM_MF2	PBS	8
AM_MF3	PBS	12
AM_MF4	MilliQ water	4
AM_MF5	MilliQ water	8
AM_MF6	MilliQ water	12
Sonication Method (SC)		Total Sonication Time (s)
AM_SC7	PBS	36
AM_SC8	MilliQ water	36
AM_SC9	MilliQ water	60
Freeze and Thawing (FT) Method		Freeze–Thawing Cycles (n°)
AM_FT10	PBS	3
AM_FT11	MilliQ water	3
AM_FT12	MilliQ water	5
Electroporation (EP)		Voltage (V)
AM_EP13	PBS	100
AM_EP14	PBS	200
AM_EP15	PBS	300
AM_EP16	MilliQ water	100
AM_EP17	MilliQ water	200
AM_EP18	MilliQ water	300

**Table 5:** Composition of empty and BSA-loaded charged liposomes obtained through passive (microfluidic) and active (freeze–thawing) methods.

Batch	DSPC:Chol	DOTAP	PE	DOPS	BSA (10 mg/mL), Trehalose (20% *w*/*w*)	Passive Method	Active Method
PM3	49.5:49.5	1	-	-	-	X	-
PM4	49.5:49.5	1	-	-	X	X	-
PM5	47.5:47.5	5	-	-	-	X	-
PM6	47.5:47.5	5	-	-	X	X	
AM_FT19	47.5:47.5	5	-	-	X		X
PM7	49.5:49.5	-	1	-	-	X	-
PM8	49.5:49.5	-	1	-	X	X	-
PM9	47.5:47.5	-	5	-	-	X	-
PM10	47.5:47.5	-	5	-	X	X	-
AM_FT20	47.5:47.5	-	5	-	X		X
PM11	49.5:49.5	-	-	1	-	X	-
PM12	49.5:49.5	-	-	1	X	X	-
PM13	47.5:47.5	-	-	5	-	X	-
PM14	47.5:47.5	-	-	5	X	X	-
AM_FT21	47.5:47.5	-	-	5	X		X

X states for selected for composition and method indicated in Table head; - states for not selected for composition and method indicated in table head.

## Data Availability

Not applicable.

## Supplementary Materials

The following supporting information can be downloaded at: https://www.mdpi.com/article/10.3390/ijms241713542/s1.
