# Supplementary material for: Investigation and Comparison of Active and Passive Encapsulation Methods for Loading Proteins into Liposomes
Source: Int J Mol Sci. 2023 Aug 31;24(17):13542. doi: 10.3390/ijms241713542 (PMC10487800; doi:10.3390/ijms241713542)
Supplement: Supplementary file 1 [file ijms-24-13542-s001.zip › ijms-2552364-supplementary.pdf]

SUPPLEMENTARY DATA

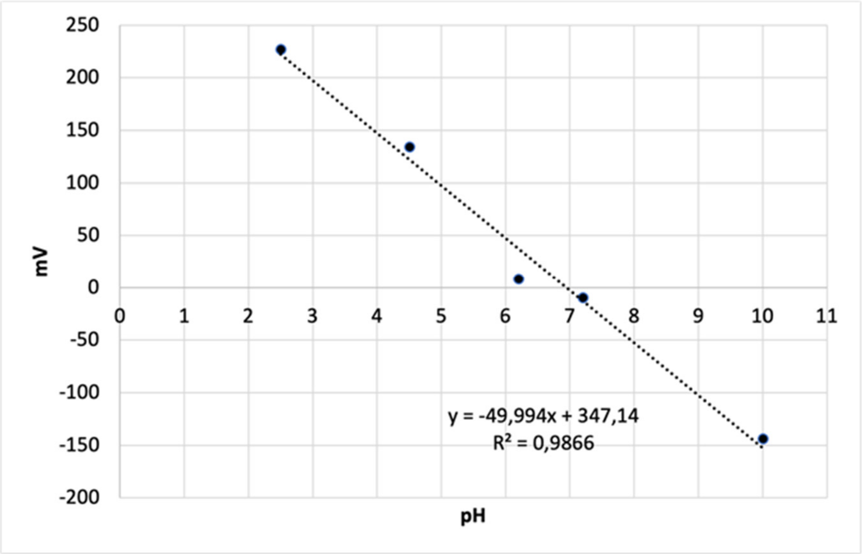

Figure S1. PE:DSPC:Chol liposomes surface charge in different aqueous media, as evaluated on batch #PM9.

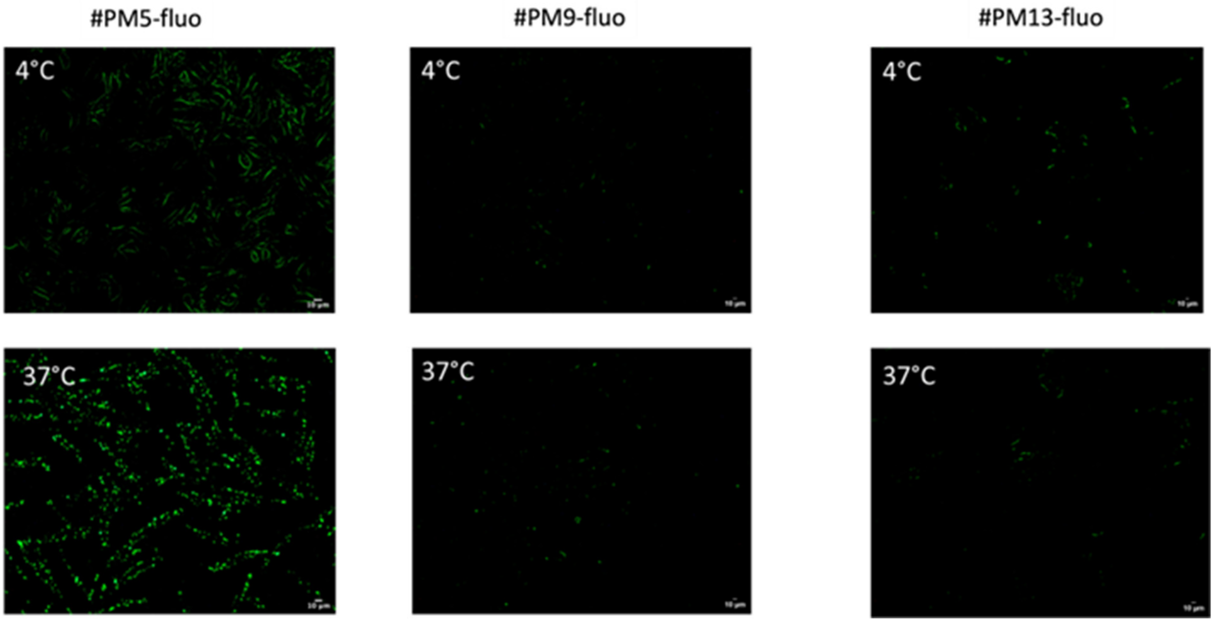

Figure S2: cellular uptake 10X of batches #PM5-fluo, #PM9-fluo and #PM13-fluo after 4h at 4°C and 37°C.
